# Drying anti-malarial drugs *in vitro* tests to outsource SYBR green assays

**DOI:** 10.1186/s12936-015-0600-z

**Published:** 2015-02-21

**Authors:** Karim Traore, Adeline Lavoignat, Guillaume Bonnot, Fatimata Sow, Giuliana C Bess, Marjorie Chavant, Frederick Gay, Ogobara Doumbo, Stephane Picot

**Affiliations:** Malaria Research and Training Center, DEAP/FMPOS, UMI3189, Université des Sciences, des Techniques et des Technologies de Bamako, BP 1805 Bamako, Mali; Malaria Research Unit, SMITH, ICBMS, UMR 5246 CNRS-INSA-CPE-University Claude Bernard Lyon1, 8 Avenue Rockefeller, 69373, Lyon, Cedex 08 France; Institut de Parasitologie et Mycologie Médicale, Hospices Civils de Lyon, Lyon, France; AP-HP, Service de Parasitologie-Mycologie, Université Pierre et Marie Curie Paris 6, Paris, France

**Keywords:** Malaria, SYBR green, *In vitro*, *Ex vivo*, Drug resistance

## Abstract

**Background:**

Measurement of anti-malarial drug efficacy and resistance relies mainly on *in vivo* clinical trials, *in vitro/ex vivo* assays and molecular markers detection. The existing *in vitro/ex vivo* assays, in particular those that are using non-radioactive devices, need to be standardized and adapted to field conditions. SYBR Green assay offers a rapid and cheap alternative to other *in vitro* assays, but it requires tools not commonly available in field laboratories. Here is described a modified SYBR green I protocol to perform the parasite growth test with blood samples in endemic areas, followed later by the SYBR green fluorescence assay performed at a specialized laboratory level.

**Methods:**

*In vitro* susceptibility of *Plasmodium falciparum* clones HB3, 3D7, W2 and 7G8 to chloroquine (CQ), dihydroartemisinin (DHA), pyronaridine (PYD) and piperaquine (PPQ) was tested. Fresh isolates of *P. falciparum* from imported malaria cases were collected for *ex vivo* assays. The parasite suspension was added in 96-well plates predosed with anti-malarial drugs and incubated for 72 hours at 37°C, 5% CO2. SYBR green I protocol was modified to dry the plates after freeze-thawed process to mimic storage and shipping conditions. The plates were rehydrated with 200 μl of complete RPMI medium for fluorescence assay.

**Results:**

There were no significant differences in IC_50_ values of CQ, DHA, PYD and PPQ, determined by the modified protocol, compared to standard protocol. Longer storage did not affect the IC_50_ values.

**Conclusion:**

The SYBR green I modified protocol produced reliable results and could be a suitable method for *in vitro/ex vivo* assays in field.

## Background

Malaria kills many children annually and is endemic in more than 100 countries [1,2]. The spread of resistant malaria parasites to anti-malarial drugs, including artemisinin-based combination therapy, is a major concern for malaria control in endemic countries [3]. In the absence of effective vaccine, chemotherapy remains one of the strategies to control malaria. Continuous monitoring of current anti-malarial drugs is needed to detect the spread of resistant parasites strains and estimate the risk of therapeutic failure.

Three main approaches including *in vivo* trials, *in vitro/ex vivo* assays and molecular markers of drug resistance are currently used to monitor anti-malarial drug efficacy and drug resistance [4]. *In vivo* studies are considered the gold standard for measuring the efficacy of anti-malarial drugs [5]. They allow the measurement of clinical and parasitological efficacy of anti-malarials drugs and the detection of changes in treatment outcomes. Carrying out *in vivo* efficacy trials requires time, qualified medical staff and financial resources, which can represent a challenge in resource-limited malaria-endemic countries. In addition, *in vivo* trials measure therapeutic efficacy rather than drug resistance [6,7].

*In vitro/ex vivo* assays are indispensable means for measuring the intrinsic susceptibility of malaria parasites to anti-malarial drugs, and establishing baseline susceptibility of local parasite isolates to newly introduced drugs. Several techniques for *in vitro/ex vivo* tests have been developed for decades. These techniques include the WHO microtest, the isotopic based tests, the enzyme-linked immunosorbent assay (ELISA) and the SYBR green-based tests [8,9]. However, the availability of these techniques is limited in low-income countries due to financial cost, time consumption and problems related to the management of radioactive waste. The existing *in vitro/ex vivo* assays, in particular those that are non-radioactive, need to be standardized [8]. SYBR Green I *in vitro/ex vivo* assay offers a rapid, reproducible and cheap alternative to radioisotopic methods [4,10,11]. This also requires specific material and expertise commonly not available in field laboratories.

Here is the report of a modified SYBR green I protocol to perform *in vitro/ex vivo* tests in two steps: doing parasite culture on 96-well plate containing anti-malarial drugs in standard conditions, and postponing the step of SYBR green fluorescence assay for outsourcing with specialized laboratories. Such a standardized protocol for conducting *in vitro*/*ex vivo* drug sensitivity assays for field monitoring of drug-resistant malaria would allow direct comparisons of *in vitro/ex vivo* results from different laboratories involved in networks of anti-malarial drugs testing. It would also allow collecting more data during field studies.

## Methods

### Anti-malarial drugs

The following drugs were tested for *ex vivo* and *in vitro* assay: chloroquine diphosphate salt (CQ, RMCQ20120605-03 [20 mg]); dihydroartemisinin (DHA, RMDHA20120511-04 [20 mg]); pyronaridine tetraphosphate (PYD, RMPD20130114-01 [200 mg]) and piperaquine tetraphosphate tetrahydrate (PPQ, RMPQ20130114-01 [200 mg]). These drugs were provided by the WorldWide Antimalarial Resistance Network (WWARN). The stock solutions of CQ, DHA and PPQ were prepared in sterile distilled water and DHA in methanol. A two point five–fold serial dilution of stock solutions was made to obtain seven different concentrations. The final concentrations ranged from 6.55 nM to 1600 nM for CQ, 0.07 nM to 16 nM for DHA, 0.33 nM to 80 nM for PYD and 1.31 to 320 nM for PPQ (Table 1). Drugs were distributed at 25 μl per well in 96-wells plates. Each drug was distributed in triplicate in the plate. The plates for *ex vivo* tests were dried overnight at room temperature in sterile conditions and sealed with an adhesive plastic, then stored at +4°C until use.

**Table 1:** **The final concentrations of drugs in serial dilutions for** ***in vitro*** **/** ***ex vivo*** **assay**

**Dilution (nM) of Drugs**	**D1**	**D2**	**D3**	**D4**	**D5**	**D6**	**D7**	**D8**
CQ	1600	640	256	102	41	16.4	6.55	0
DHA	16	6.4	2.56	1.02	0.41	0.16	0.07	0
PYD	80	32	12.8	5.12	2.05	0.82	0.33	0
PPQ	320	128	51.2	20.5	8.19	3.28	1.31	0

CQ = chloroquine; DHA = dihydroartemisinin; PPQ = piperaquine; PYD = pyronaridine. nM = nanomolar. All drugs concentrations are in nM.

### SYBR green I *in vitro/ex vivo* modified protocol

#### *In vitro* assay

**Figure 1:**
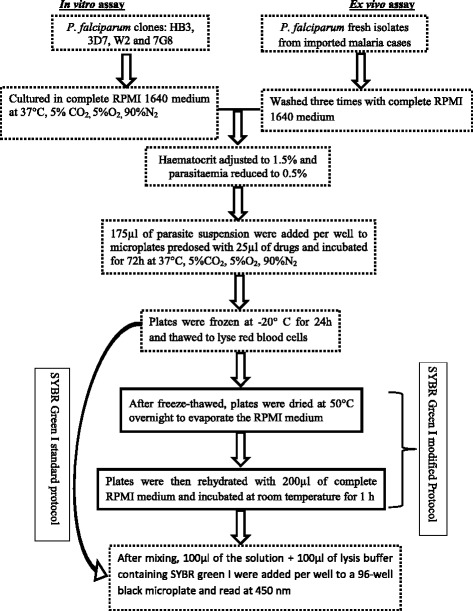
**SYBR Green I modified protocol for** ***ex vivo/in vitro*** **assay.**

Two chloroquine-sensitive (HB3, 3D7) and two chloroquine-resistant (7G8, W2) clones of *Plasmodium falciparum* were tested for *in vitro* susceptibility against chloroquine, dihydroartemisinin, pyronaridine and piperaquine. 3D7 and W2 were provided by WWARN; HB3 and 7G8 were from The Malaria Research and Reference Reagent Resource Center (MR4). The clones were cultured in complete RPMI 1640 medium, with 0.5% Albimax + hypoxanthine and gentamicin. They were incubated at 37°C, 5%CO2, 5%O_2_, 90%N_2_. An aliquot of the culture was diluted to reduce the parasitaemia to 0.5%, and the haematocrit was adjusted to 1.5%. This suspension was then added (175 μl per well) to microplates predosed with 25 μl of drugs and incubated for 72 hours at 37°, 5%C0_2_, 5%O_2_, 90%N_2_. After freeze-thawed process, the test was performed according to the SYBR Green I standard and modified protocols (Figure 1). For the standard protocol, the test was performed as described by Bacon et al. [10]. Briefly, after homogenization of the medium, lysis buffer and SYBR Green I were added and fluorescence was read with the fluorimeter Tristar2 (Multimode Reader LB 942 Bertold Technologies) at 485 nm wave length using Microwin 2000 software. The standard protocol was then modified by drying the plates at 50°C for 10 hours after freeze-thawed process to evaporate the culture medium (Figure 1). To assess the impact of storage period on the values of IC_50,_ the dried plates were stored at room temperature during 24 hours and nine days. The plates were then rehydrated with 200 μl of complete RPMI 1640 medium and incubated at room temperature for at least 1 hour. Lysis buffer and SYBR Green I were added and fluorescence were read with the fluorimeter Tristar2 (Multimode Reader LB 942 Bertold Technologies) at 485 nm wave length using Microwin 2000 software. One millilitre of the suspension was frozen at -20°C as control for parasite growth.

#### *Ex vivo* assay

Fresh isolates were collected from nine imported clinical malaria cases with parasitaemia ≥ 0.5% at the Lyon teaching hospital, France. Tests were done within 48 hours after bleeding, without culture adaptation. Blood samples were collected before any anti-malarial treatment, washed three times in complete RPMI 1640 medium and adjusted to 1.5% haematocrit and 0.5% parasitaemia by adding fresh red blood cells. 175 μl suspension was added per well to the plates containing 25 μl of the anti-malarial drugs. Plates were incubated for 72 hours at 37°C in 5% CO2 and O_2_ controlled atmosphere. After the incubation time, plates were frozen and thawed for red blood cells lysis before SYBR Green I analysis. SYBR Green I analysis was performed according to the standard and modified protocols as already described above for *in vitro* tests (Figure 1).

### Statistical analysis

The results from the Tristar2 were given in fluorescence units (FU). The concentration at which the drugs were able to inhibit 50% of parasite growth (IC_50_) was calculated through non-linear regression software. IC_50_ values were validated if there was sufficient growth of parasites with the ratio of FU (FU at concentration 0/FU at maximum concentration) ≥ 2, and a sigmoid curve generated by the software. IC_50_ means were compared using the *t*-test (α = 0.05) and the Ficher-Snedecor F-test for variances.

## Results

Overall, three *in vitro* tests were performed for each clone. The IC_50_ means calculated respectively by standard and modified protocols are summarized in Table 2. No significant differences were seen in IC_50s_ generated for *in vitro* tests between the standard and modified protocols.

**Table 2:** **The means of CI**_**50**_ **of the SYBR green based** ***in vitro*** **anti-malarial drugs assay according to protocols**

**Clone/drugs**	**Protocol standard**		**New Protocol (test)**		**P**
	**Mean CI**_**50**_ **(nM)**	**95% CI**	**Mean CI**_**50**_ **(nM)**	**95% CI**	
3D7					
CQ	31.10 ± 6.02	24.28-37.91	32.43 ± 4.31	26.22-35.97	0.77
DHA	2.30 ± 0.68	1.53-3.06	1.93 ± 0.43	1.81-2.78	0.47
PYD	8.82 ± 1.17	7.49-10.14	8.89 ± 1.54	7.07-10.56	0.97
PPQ	26.33 ± 1.71	21.38-28.27	25.66 ± 6.82	15.61-34.4	0.88
W2					
CQ	467.66 ± 51.18	409.74-525.57	500.66 ± 87.22	368.96-566.35	0.60
DHA	1.94 ± 1.03	0.77-3.10	1.81 ± 0.99	0.81-3.06	0.81
PYD	5.35 ± 1.27	3.90-6.79	4.33 ± 1.21	3.98-6.71	0.37
PPQ	19.50 ± 2.91	16.20-22.79	17.20 ± 3.14	15.94-23.05	0.40
HB3					
CQ	37.92 ± 6.66	30.38-45.47	50.28 ± 16.77	31.30-69.26	0.33
DHA	2.10 ± 0.97	0.99-3.20	1.99 ± 0.41	1.53-2.46	0.87
PYD	11.78 ± 2.36	9.10-14.46	12.51 ± 3.40	8.65-16.36	0.70
PPQ	21.70 ± 1.76	19.70-23.70	18.51 ± 2.24	15.97-21.06	0.12
7G8					
CQ	416.98 ± 92.57	311.82-521.33	420.55 ± 96.04	311.87-529.24	0.18
DHA	0.65 ± 0.17	0.45-0.85	0.73 ± 0.12	0.58-0.87	0.56
PYD	4.43 ± 1.39	2.84-6.01	5.05 ± 0.34	4.66-5.44	0.52
PPQ	17.82 ± 3.57	13.77-21.87	15.98 ± 1.22	14.59-17.38	0.47

CQ = Chloroquine; DHA = Dihydroartemisinin; PPQ = Piperaquine; PYD = Pyronaridine; SD = Standard deviation, nM = nanomolar.

*Ex vivo* susceptibility of *P. falciparum* was tested for nine samples from patients. The average culture success rate was 61.5% (ranged from 55.5-66.6%). The IC_50_ were calculated respectively by standard and modified protocols and the results are summarized in Table 3. No significant differences were seen in IC_50s_ generated for *ex vivo* samples between the standard and modified protocols.

**Table 3:** ***Ex vivo*** **susceptibility of** ***Plasmodium falciparum*** **isolates to chloroquine, dihydroartemisinin, pyronaridine and piperaquine (standard and modified protocols)**

	**Culture success rate (n/N)**	**Standard protocol**	**Modified protocol**	***P***
		**IC**_**50**_ **mean (nM) [95% CI]**	**IC**_**50**_ **mean (nM) [95% CI]**	
CQ	66.6% (6/9)	484.08 [176-791]	730.66 [595-995]	0.35
DHA	66.6% (6/9)	1.67 [1.21-2.12]	1.44 [0.88-2]	0.55
PYD	55.5% (5/9)	14.23 [2.83-25.63]	16.85 [1.69-32]	0.79
PPQ	55.5% (5/9)	78.22 [36.56-119.87]	74 [24.61-123.38]	0.90

CQ = Chloroquine; DHA = Dihydroartemisinin; PPQ = Piperaquine; PYD = Pyronaridine, nM = nanomolar.

## Discussion

Among the different *in vitro/ex vivo* assay methods, the use of fluorescent labeling of DNA with SYBR Green offers some advantages compared to methods that use tritiated hypoxanthine or ELISA [4,10-12]. However, the fluorescence determination requires material not commonly available in field laboratories. Postponing the step of fluorescence determination allows the use of SYBR Green I protocol in field laboratories with limited resources. It also offers opportunity (i) to do simultaneously *ex vivo* and *in vivo* tests during field studies, (ii) to standardize protocols for data collection during multicenter clinical studies, (iii) to store data for future use, (iv) and to share original data with collaborators. The SYBR Green I modified protocol described in this study enables to dry and store the plates, which will be analysed later by well-equipped laboratories. Networks devoted to anti-malarial drugs resistance assay [13-17], may use this modified protocol for multicentre clinical studies, that could result in reduction of instrumental and systematic bias and facilitate the compilation, analysis and interpretation of data from different study sites. The risks of environment pollution are scarce and the technique does not require specific protection of manipulator since the SYBR Green is not radioactive [8] while its toxic for DNA, and will be added to the culture in the laboratories where the fluorescence will be read.

The plates were dried at 50°C to minimize the risk of contamination of the culture with bacteria or fungi (higher temperatures could eliminate the risk of contamination, but they could denature the DNA of the parasite). The modified protocol was first tested with laboratory clones *in vitro*, then with field fresh isolates *ex vivo*. Samples were collected from nine imported malaria cases (six from Cameroon and three from the Democratic Republic of Congo). Four patients have declared the use of chemoprophylaxis against malaria (with chloroquine or chloroquine-proguanil) prior to admission. The average of culture success rate (interpretable tests) of *ex vivo* assays was 61.5%, slightly lower than this described by Tinto *el al.* (85%) [18], probably due to the small sample size of the study reported here.

There was no significant difference between the means of IC_50_ determined by the standard and modified protocols. The values of IC_50_ determined *in vitro* by the modified protocol were broadly similar to those calculated using the standard protocol (p > 0.10). The duration of storage did not affect the IC_50_ value (IC_50_ of CQ against 7G8 ranged from 676 nM in standard protocol to 502 nM after 24 hours and 9 days of storage of dried plates in modified protocol). A slight but not significant decrease was observed in IC_50_ values of dried plates. These findings suggest that the dried plates can be stored at room conditions and be sent without special transportation requirements since biological material is stable and non-infectious.

The means of IC_50_ of chloroquine against sensitive clones 3D7 (32.43 nM, 95% CI: 26.22-35.97) and HB3 (50.28 nM, 95% CI: 31.3-69.2) determined *in vitro* by the modified SYBR Green I protocol are broadly similar to those already described *in vitro* by Garbi et al. in Senegal, Mali, Cameroon and Côte d’Ivoire [19] and Issaka et al. in Niger [20]. For *ex vivo* assays, our IC_50_ values of CQ (484.08 and 730.66 nM) are also comparable to the maximum values described by Tinto et al. (using the semiautomated microdilution technique) in Burkina Faso (8.3-595.9 nM) [18]. The lowers values (8.3 nM) obtained by Tinto et al. could be the fact that resistant and sensitive strains are circulating simultaneously in populations, and the large sample size included in this study.

The means of IC_50_ of the chloroquine were high in *ex vivo* assays (484.08 and 730.66 nM respectively in standard and modified protocols). Malaria cases were imported from chloroquine-resistance areas. High resistance rates of *P. falciparum* to chloroquine have been described in Cameroon since 2000 [21], even though recent studies have shown decreases in resistance due to changes in malaria treatment policies in these areas [19].

## Conclusion

Drying and storing the plates did not affect the means of IC_50_ values in a modified SYBR Green I protocol to assess anti-malarial drugs. The modified protocol allows performing *in vitro/ex vivo* tests in two steps. By postponing the step that requires SYBR Green methodology, the tests could be done in field conditions and the dried plates could be transported in ambient conditions. Field staff involved in multicentre studies may use this protocol to standardize the collect and share of data. It offers the possibility to do simultaneously clinical *in vivo* and *ex vivo/in vitro* assays in field. More *in vitro*/*ex vivo* studies with large sample size are necessary for the validation of this protocol.

## Abbreviations

CQ: Chloroquine
DHA: Dihydroartemisinin
ELISA: Enzyme-linked immunosorbent assay
FU: Fluorescence units
GIS: Geographic information system
MR4: Malaria research and reference reagent resource center (MR4)
IC_50_: The 50% inhibitory concentration
nM: Nanomolar
PPQ: Piperaquine
PYD: Pyronaridine
RPMI: Roswell park memorial institute
SD: Standard deviation
WANECAM: West african network for clinical trials of antimalarial drugs
WHO: World health organization
WWARN: Worldwide antimalarial resistance network
